# Sublingual Misoprostol versus Oxytocin to Induce Labor in Term Premature Rupture of Membranes in Pregnant Women: A Randomized Single-Blind Controlled Trial

**DOI:** 10.1155/2022/9449036

**Published:** 2022-02-13

**Authors:** Suchada Unthanan, Kanyarat Petcharat, Sinart Prommas, Buppa Smanchat, Kornkarn Bhamarapravatana, Komsun Suwannarurk

**Affiliations:** ^1^Department of Obstetrics and Gynecology, Bhumibol Adulyadej Hospital, Royal Thai Air Force, Bangkok, Thailand; ^2^Department of Preclinical Sciences, Faculty of Medicine, Thammasat University, Pathumthani, Thailand; ^3^Department of Obstetrics and Gynecology, Faculty of Medicine, Thammasat University, Pathumthani, Thailand

## Abstract

**Objective:**

The aim of this study was to compare maternal and neonatal outcomes between sublingual misoprostol and oxytocin on stimulating labor in term premature rupture of membranes (PROM) in pregnant women. *Materials and method*. This randomized single-blind control trial was conducted at Bhumibol Adulyadej Hospital (BAH), Royal Thai Air Force, Bangkok, Thailand, between September 2020 and February 2021. Subjects were term pregnant women who had PROM and came to BAH for delivery. Participants were allocated into study (misoprostol) and control (oxytocin) groups. The study and control groups were, respectively, administered sublingual misoprostol and intravenous oxytocin to induce labor. Induction time and second stage of labor were recorded. Neonatal outcomes and maternal and fetal complications were also recorded and analyzed.

**Result:**

A total of 170 women were enrolled and equally divided into study and control groups. Mean maternal age, body mass index, parity, gestational age, and bishop score of both groups were comparable. Induction time of the study group was statistically shorter than the control group (338 and 399 min, respectively). Duration of active phase (450/427 min) and the second stage (19/21 min) of labor between study and control groups were not significantly different. Cesarean section delivery rate of study was lower than the control group (13.3 and 28.8%, *p* = 0.002). Intrapartum complications, neonatal outcomes, and intra- and postpartum complications among both groups were not significantly differentiated. There was no instance of postpartum hemorrhage or uterine rupture in the present study.

**Conclusion:**

Induction time and cesarean section rates of sublingual misoprostol group were significantly lower than the intravenous oxytocin group in full-term PROM pregnancy.

## 1. Introduction

Premature rupture of membranes (PROM) is a spontaneous leakage of amniotic fluid from the amniotic sac before the second stage of labor. It is one of the most problematic conditions leading to complications in both mother and newborn. The incidence of PROM was around 8-10% in term pregnancy with an especially high rate in developing countries [1]. Maternal and neonatal infections occur alarmingly frequently in PROM cases. Prolonged duration of amniotic membrane ruptures or leakages led to high risk of maternal and neonatal infection [1].

Sixty percent or more of term pregnant women presented with 20% chorioamnionitis within 24 hours of PROM. Chorioamnionitis resulted in 2.3 to 3.4 times relative risk of maternal morbidity (septic pelvic vein thrombophlebitis, and pelvic abscess) increased risk for metritis and puerperal infection [2]. PROM duration greater than 24 hours PROM resulted in a 14% increased risk for significant maternal morbidity, namely, from sepsis, transfusion, hemorrhage, infection, acute renal injury, and readmission [3].

As a result of decreased amniotic fluid, the newborn of PROM mother was at increased risk for umbilical cord compression, congenital anomaly, and other complications which may be lethal. PROM in term pregnant women was one of the most recommended reasons for labor induction to avoid maternal and neonatal morbidity [1].

Oxytocin has been an agent of choice for labor induction. It allowed adequate uterine contraction resulting in shorter duration to delivery. However, the oxytocin route of administration was mainly intravenous. As a result, oxytocin administration required special medical attention both for dose management and a look out for possibility of further infection.

Misoprostol (prostaglandin E1) has been introduced in labor induction since 2007. Oral and vaginal routes of administration were both explored. Previous literature reported satisfaction in the mechanical aspects of misoprostol as uterine contraction inducing agent [4].

However, efficacy of both oral and vaginal route of misoprostol administration depended heavily on the medical personnel who prepared the oral solution or uncertainty of proper insertion of vaginal suppository [4]. Both of which were human factors which could not be easily controlled.

This study is aimed at comparing misoprostol usage to oxytocin for induction of labor. Obstetrics outcomes, namely, labor time, maternal, and newborn outcomes, would be compared.

## 2. Materials and Methods

This randomized single-blind control trial was conducted at the Department of Obstetrics and Gynecology, Bhumibol Adulyadej Hospital (BAH), Thailand. It was reviewed, and ethical approval was granted by the institutional review board (TCTR20200722003).

Subjects were pregnant women who came for delivery at BAH during the period between September 2020 and February 2021. Some attended antenatal care at BAH but some were walk-ins for delivery cases. Subjects who had ruptured membranes and fit our criteria were approached. The scope of study was explained, and women who participated all signed informed consent forms.

The inclusion criteria of the study were term singleton pregnancy, PROM, cephalic presentation, parity less than 5, no history of hypersensitivity to prostaglandins, candidate for vaginal delivery, bishop scores ≤ 6 (unripped cervix), and no uterine scarring. Exclusion criteria were abnormal fetal heart rate patterns, abnormal clinical pelvimetry and labor progression, prior uterine surgery, and refusal to participate in the study. Membranes were clinically confirmed by visualization of pooling fluid in the posterior vaginal fornix through the cervix during sterile speculum examination.

Gestational age (GA) was calculated based on the first or early second trimester ultrasonography. Ultrasonography was performed to survey the obstetric information, namely, position, placenta location, amniotic fluid index, fetal viability, and estimated fetal weight upon arrival in walk-in delivery cases. Digital examination of cervix was determined to assess cervical dilation, effacement, station, position and cervical consistency.

Enrolled patients were then allocated to study or control groups according to computer generated simple random assignment in concealed envelopes by SPSS software version 22 for Mac (SPSS, Inc., Chicago, IL). Participants in both study and control groups received sublingual misoprostol and intravenous oxytocin infusion for induction of labor, respectively. The participants did not know the allocation process.

According to Sahin et al., mean induction to delivery time among two populations was calculated for appropriate sample size [5]. Mean difference of induction time, alpha, and beta errors between misoprostol and oxytocin groups was set at 0.96, 0.05, and 0.2, respectively. Statistical analysis was performed using the SPSS, version 17 (SPSS Inc, Chicago, USA). The sample size was 85 cases per group from calculation. The total participants in this study were 170 cases for drop out compensation.

Participants in the study group received 25 *μ*g sublingual misoprostol every two hours as needed up to a total of six maximum allowable doses. The 25 *μ*g misoprostol was prepared by a BAH pharmacist using a pill cutter. Amount of sublingual misoprostol doses required by each subject until their delivery was recorded. Any subject who had not delivered at 12 hours after initial misoprostol administration would be considered a failed case but continue to receive proper delivery care for safe delivery. In the control group, oxytocin was administered by continuous IV infusion with a use of a controlled infusion pump beginning at 2 mU/min. The dose was increased by 2 mU/min every 20 minutes until adequate contractions were achieved at 4-5 contractions every 10 minutes.

All concentrations and doses of oxytocin required for each subject were recorded. Any patients in the control group who had no successful delivery at the end of 12 hours from the initiation of IV oxytocin administration would be considered a failed case but would continue to receive proper care for her delivery (as needed) by the attending obstetricians.

Fetal heart rate (FHR) was continuously monitored during the induction of labor to diagnose any abnormal fetal monitoring. Appropriate treatment would be initiated according to FHR category tracing if needed. Active labor was defined as regular uterine contractions and cervical dilation of >5 cm. Tachysystole was defined as >5 contractions within 10 minutes for two consecutive 10-minute periods. Uterine hyperstimulation syndrome was defined as any FHR decelerations or other varying FHR changes combining with uterine contraction longer than 90 seconds or having more than or equal to 6 times of contractions in 10 minutes. Conservative management was the first option for these abnormalities (left lateral positioning, oxygen therapy, discontinuation of oxytocin infusion, hydration with 500 cc Ringer lactate for 15 minutes). If there was no improvement in abnormality with conservative management, the next steps would be determined by the FHR abnormality category [6].

Prophylactic antibiotics were administered to prevent neonatal sepsis in cases in which the PROM lasted >18 hrs or if the mother's body temperature was >38 degree Celsius and suspected to represent chorioamnionitis [3]. Failed induction was defined as an inability to generate regular contractions and cervical changes with either misoprostol or oxytocin administration for more than 12 hours after starting induction in both groups.

In both groups, cesarean section delivery was performed when standard obstetric indications were presented. All participants in both groups were monitored for signs of labor and vital signs. Continuous electronic fetal heart rate monitoring was applied to all cases.

The primary outcome measured was the induction to delivery interval (the time from first dose of misoprostol or start of oxytocin to vaginal delivery). Secondary outcomes were duration of second stage of labor, hyperstimulation, failed induction rates, and neonatal outcomes, namely, rate of low Apgar scores, presence of meconium, and neonatal intensive care unit admission.

All statistical analyses were performed using SPSS software version 22 for Mac (SPSS, Inc., Chicago, IL). Normal distribution data was compared via Student *t*-test, and nonnormal distribution data were compared by Mann–Whitney *U* test as appropriate. Chi-square was used to compare categorical data resulting in relative risk and 95% CI. A *p* value <0.05 was considered statistically significant.

## 3. Results

**Figure 1:**
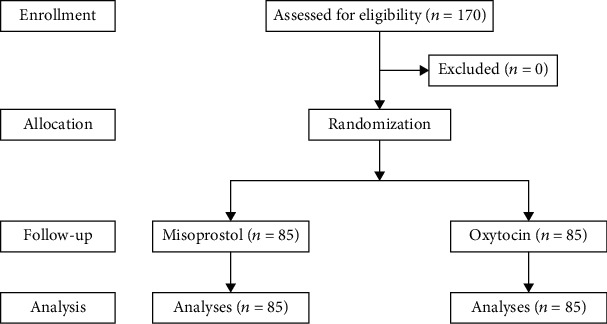
Flow chart of study.

A total of 170 women were enrolled in the study. Participants were equally divided into study and control groups as shown in Figure 1. Mean age of cases was 27.6 years old. One-fifth of patients (34/170) were nulliparity. Maternal age, prepregnancy body mass index (BMI), gestational age, parity, and bishop scores of both groups were comparable.

Duration A and all stages of labor of both groups were equally as presented in Table 1. Duration B of the misoprostol group was statistically shorter than the oxytocin group. Three-quarters of patients (73/170) had successful vaginal delivery.

There was no significant difference of obstetric outcomes, namely, estimated blood loss and intra- and postpartum complications among both groups. There were 4/9 and 2/6 cases of uterine hyperstimulation and tachysystole in misoprostol/oxytocin groups, respectively, without statistical difference. Chorioamnionitis in misoprostol and oxytocin groups was one and three cases, respectively (*p* value = 0.364). There was no uterine rupture and postpartum hemorrhage (PPH) case. Neonatal outcomes, namely, neonatal weight, Apgar score, and neonatal intensive care unit admission were similar in each group (Table 2).

## 4. Discussion

This study compared the time from induction to the active phase of labor between sublingual misoprostol and oxytocin groups. The time in the active phase and second stage of labor in the misoprostol group was significantly shorter than in the oxytocin group. The use of misoprostol for augmentation of labor in pregnant women with PROM had been an interesting research topic with different doses and preparations of prostaglandin being used. An oral preparation of 25 *μ*g misoprostol delivered every 2 hours was utilized [7, 8]. Pourali et al. [9] used 25 *μ*g SL misoprostol every 4 hours. Pourali and coworkers reported from Iran in 2017. Time from induction to active phase of labor in their work was not different among participants in the misoprostol and oxytocin groups. Patients in the misoprostol group had shorter time in the active phase and second stage of labor than the oxytocin group with statistically significant difference. The current and Pourali's studies utilized the same sublingual route of misoprostol to labor induction. The current study supported Pourali's report.

Harandi and Peter reported the effect of oral misoprostol for induction of labor from Iran to Nigeria in 2013 and 2019, respectively. Dosage of oral misoprostol in both works was 25 *μ*g every 2 hours. Time from induction to fetal delivery of Harandi and Peter among the misoprostol group was shorter than in the oxytocin group. Even though time from induction to active phase of labor in the current study among misoprostol and oxytocin groups was comparable, time from induction to fetal delivery in the current study was in line with Harandi and Peter.

In another study from Iran in the year 2019, forty microgram oral misoprostol every 2 hours given in this study resulted in shorter average duration of labor compared to the result reported by Kashanian's group orally [10] given at 338 compared to 474 minutes, respectively. The current study supported Kashanian's study. Twenty five micrograms vaginal misoprostol every 6 hours in Acharaya's study resulted in shorter average time from induction to fetal delivery compared to the result reported by Ferret's group buccally given at 1074 compared to 996 minutes, respectively. The current study supported Acharaya's and Ferret's study.

The onset and duration of sublingual misoprostol were 8 and 180 minutes, respectively [11]. This misoprostol property might explain that the time from induction to the active phase of labor among both misoprostol and oxytocin groups was similar. The effect of misoprostol appeared in the first and the second stage of labor. Sublingual misoprostol was a suitable alternative to oxytocin. The duration of labor from augmentation to delivery was significantly reduced in cases receiving sublingual misoprostol as opposed to cases that received oxytocin (*p* = 0.004).

In terms of fetal outcomes, with regards to birth weight and neonatal outcomes such as Apgar scores and NICU admissions, the results observed in this study were similar to previous studies such as that of Kashanian's, Harandi's, Peter's, Acharya's, Pourali's, and Freret's [7–10, 12, 13]. However, there were no NICU admissions for all deliveries.

Uterine rupture and postpartum hemorrhage were the most catastrophic complications from prior studies. There were no cases of uterine rupture and postpartum hemorrhage observed in this study. Sublingual misoprostol was proved safe in labor induction. With regards to other complications such as chorioamnionitis which was another key complication of patients presenting with PROM, the mean time between rupture of membranes to delivery observed in this study (532 minutes) was significantly lower than in previous studies (631 minutes) [9]. However, participants in our misoprostol group had lower rates of chorioamnionitis when compared to participants in the oxytocin group as detailed in Table 2. However, this difference was not statistically significant. Other complications from augmented deliveries, either by misoprostol or oxytocin, included intrapartum complications such as hyperstimulation and tachysystole but yielded no significant results.

Limitations for this investigation included inability to totally blind the study and control groups on the physicians end due to the fact that drug prescription protocol did not allow doctors to be blinded when misoprostol was given. This study does not contain any conflicts of interest.

## 5. Conclusion

Stimulating uterine contraction in term pregnant PROM cases with sublingual misoprostol resulted in a shorter time to delivery compared to intravenous oxytocin injection.

## Figures and Tables

**Table 1:** Baseline characteristics of the sublingual misoprostol (*n* = 85) and oxytocin (*n* = 85) population.

	Misoprostol	Oxytocin	*p* value
Age (years)	27.07 ± 6.16	28.2 ± 5.87	0.305^T^
BMI (kg/m^2^)	28.27 ± 4.39	28.01 ± 4.84	0.758^T^
Gestational age (weeks)	38.60 ± 1.18	38.53 ± 1.25	0.739^T^
Nulliparity	18 (43.05)	16 (41.35)	0.728^C^
Bishop score	4.78 ± 1.11	4.98 ± 1.12	0.330^T^

BMI: body mass index; T: *t*-test; C: chi square.

**Table 2:** Maternal, fetal outcomes, and complication.

	Misoprostol	Oxytocin	*p* value	
Duration A (min)	532.05 (321-792)	547 (377-831)	0.516^M^
Duration B (min)	338 (243-514.5)	399 (312-672)	0.004^M^	
1^st^ stage of labor(min)	450 (317.50-655)	427.50 (335-595)	0.656^M^	
2^nd^ stage of labor (min)	19 (13-23.5)	21 (16-26)	0.555^M^	
3^rd^ stage of labor (min)	9 (6-12.5)	10 (8-12)	0.604^M^	
Cesarean section delivery	12 (13.33)	25 (28.81)	0.002^C^	
EBL (ml)	200 (175-300)	300 (200-350)	0.150^M^	
Birth weight (gm)	3084.67 ± 385	3059.42 ± 334.73	0.704^T^	
Apgar score				
1 min	8.93 ± 0.31	8.88 ± 0.67	0.591^T^	
5 min	9.97 ± 0.18	9.98 ± 0.13	0.572^T^	
< 7 at 1 min	0	1 (1.69)	0.496^F^	
NICU admission	0	0	—	
Intrapartum complications				
Hyperstimulation	4	9	0.003^F^	
Tachysystole	2	6	0.002^F^	
Postpartum complications				
Uterine rupture	0	0	—	
Chorioamnionitis	1	3	0.364^F^	
PPH	0	0	—	

Duration A: duration from ruptured of amniotic membranes before hospital admission; duration B: duration between start of labor induction to delivery; NICU: neonatal intensive care unit admission; EBL: estimated blood loss; PPH: postpartum hemorrhage; M: Mann–Whitney *U* test; T: *t*-test; F: Fisher exact test.

## Data Availability

Data used to support the findings of this study are available from the corresponding author request.
